# Targeting Human Telomeric G-Quadruplex DNA and Inhibition of Telomerase Activity With [(dmb)_2_Ru(obip)Ru(dmb)_2_]^4+^

**DOI:** 10.1371/journal.pone.0084419

**Published:** 2013-12-27

**Authors:** Shuo Shi, Shane Gao, Tongcheng Cao, Jie Liu, Xing Gao, Jian Hao, Chunyan Lv, Hailiang Huang, Jun Xu, Tianming Yao

**Affiliations:** 1 Department of Chemistry, Tongji University, Shanghai, China; 2 East Hospital, Tongji University School of Medicine, Shanghai, China; 3 Department of Chemistry, Jinan University, Guangzhou, China; University of Bologna & Italian Institute of Technology, Italy

## Abstract

Inhibition of telomerase by inducing/stabilizing G-quadruplex formation is a promising strategy to design new anticancer drugs. We synthesized and characterized a new dinuclear complex [(dmb)_2_Ru(obip)Ru(dmb)_2_]^4+^ (dmb = 4,4’-dimethyl-2,2’-bipyridine, obip = (2-(2-pyridyl)imidazo[4,5-f][1,10]phenanthroline) with high affinity for both antiparallel and mixed parallel / antiparallel G-quadruplex DNA. This complex can promote the formation and stabilize G-quadruplex DNA. Dialysis and TRAP experiments indicated that [(dmb)_2_Ru(obip)Ru(dmb)_2_]^4+^ acted as an excellent telomerase inhibitor due to its obvious selectivity for G-quadruplex DNA rather than double stranded DNA. In vitro co-culture experiments implied that [(dmb)_2_Ru(obip)Ru(dmb)_2_]^4+^ inhibited telomerase activity and hindered cancer cell proliferation without side effects to normal fibroblast cells. TUNEL assay indicated that inhibition of telomerase activity induced DNA cleavage further apoptosis in cancer cells. Therefore, Ru^II^ complex represents an exciting opportunity for anticancer drug design by specifically targeting cancer cell G-quadruplexes DNA.

## Introduction

**Figure 1:**
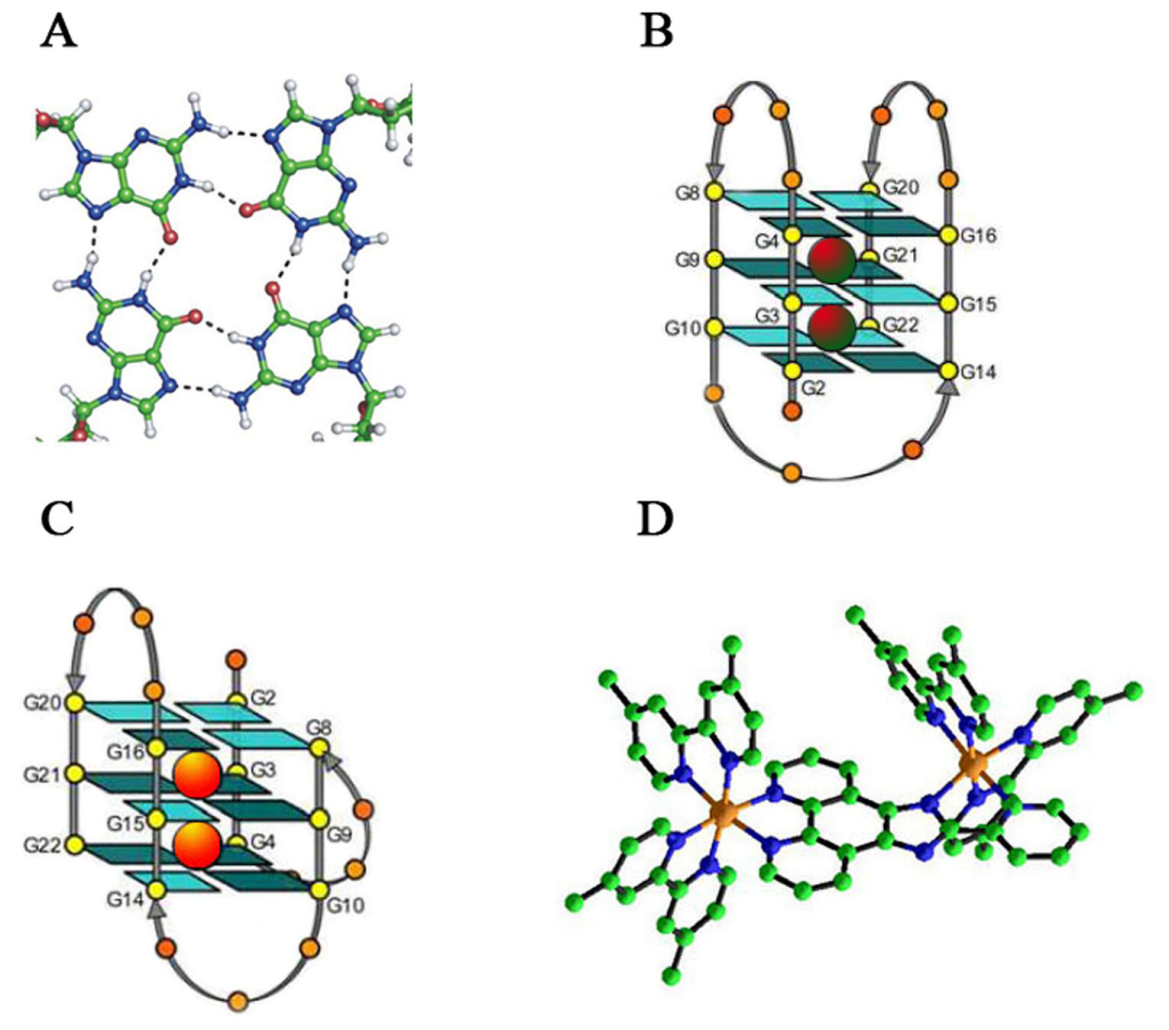
DNA and complex structures. A) structure of G-quartet with cyclic array of four guanines linked by Hoogsteen H-bonds, B) Anti-paralled G-quadruplex, C) Mixed-hybrid G-quadruplex, D)An ORTEP drawing of [(dmb)_2_Ru(obip)Ru(dmb)_2_]^4+^.

Telomeres are believed to play a vital role in genome integrity by protecting the genomic DNA from degradation and deleterious recombination events such as end-to-end fusion, rearrangements, chromosomal translocations, and chromosomal loss [1,2]. Human telomeric DNA consists of tandem repeats of double-stranded DNA sequence (5′-TTAGGG): (5′-CCCTAA) with the 3′-end capped with a 100 to 200 nucleotides single-stranded overhang [3,4]. In suitable conditions, the overhang can readily fold into a four-stranded structure known as G-quadruplex through Hoogsteen hydrogen bonds (Figure 1A). Significantly, G-quadruplex has been suggested to act as a negative regulator of telomere elongation by inhibiting telomerase activity in vivo thus considered as a potential cancer therapy target [5-10]. Meanwhile, G-quadruplex structures in gene promoter regions were also investigated as a potential new class of therapeutic targets, since the promoter regions with their diverse sequences may provide unique scaffolds ideally for designing selective ligands [11,12]. Successful forming and stabilizing in vivo G-quadruplex may be an ideal strategy to inhibit telomere elongation and telomerase activity. Besides, some important G-quadruplexes formed by human telomeric RNA sequences were also proved to be attractive therapeutic targets [13-15]. In contrast to duplex structures, G-quadruplexes show a high degree of polymorphism in terms of topological features including individual strand orientation and loop connectivity. For example, the NMR structure of 5′-AG3[T2AG3]3-3′ (denoted 22AG) in the presence of Na^+^ was an antiparallel basket quadruplex (Figure 1B) [16], but the X-ray structure for the same sequence in the presence of K^+^ revealed a parallel propeller quadruplex [17]. Furthermore, circular dichroism studies indicated that it favored a mixed parallel / antiparallel structure in the presence of K^+^ solution (Figure 1C) [18,19]. 

Hitherto, a steady growth of molecules has been reported to selectively promote the formation and / or stabilization of G-quadruplex structures for targeting telomere or telomerase. Compared with many reported organic molecules binding to G-quadruplex, metal complexes have only recently attracted systematic investigation [20-24]. Being electropositive, optical, capable to modular and facile synthesis, metal complexes take great advantages over their organic counterparts. Therefore metal complexes offer an ideal platform for sharp and rational drug design leading to easy structure and function correlation studies [20-24]. Many reports indicated that G-quadruplexes could be detected at telomeres in vivo [25-28]. Deprotection of telomeres has been shown to cause DNA damage and further induce ataxia telangiectasia mutated (ATM) dependent cell apoptosis [29-32]. Therefore, investigating the interaction between metal complexes and their targeted DNA is important for screening and developing therapeutic anticancer agents. These G-quadruplexes DNA binders have emerged as an increasingly important type of compounds in searching for novel telomerase inhibitors and anti-cancer drugs.

Herein, we reported a new dinuclear complex [(dmb)_2_Ru(obip)Ru(dmb)_2_]^4+^ (Figure 1D) with remarkable ability to specifically recognize human telomere derived G-quadruplex and inhibit telomerase activity in human cervical cancer Hela cells. As a result, our data showed this complex can inhibit the proliferation of HeLa cells and induce apoptosis, while the title complex can’t inhibit the proliferation of the human normal fibroblast cells. These promising data suggest that this dinuclear complex have great potential for anti-cancer application in vivo. 

## Experimental Section

### Materials

[Ru(dmb)_2_Cl_2_].2H_2_O [33] (dmb = 4,4’-dimethyl-2,2’-bipyridine) and obip (2-(2-pyridyl)imidazo[4,5-f][1,10]phenanthroline) [23] were prepared according to literature procedures. DNA oligomers 5′-AG3[T2AG3]3-3′ was purchased from Sangon (Shanghai, China) and used without further purification. Concentrations of these oligomers were determined by measuring the absorbance at 260 nm after melting. Single-strand extinction coefficients were calculated from mononucleotide data using a nearest-neighbor approximation. The formations of intramolecular G-quadruplexes were carried out as follows: the oligonucleotide samples, dissolved in different buffers ((a) 10 mM Tris-HCl in 100 mM Na^+^ buffer, (pH 7.4); (b) 10 mM Tris-HCl in 100 mM K^+^ buffer, (pH 7.4)), were heated to 90 °C for 5 min, gently cooled to room temperature, and then incubated at 4 °C. The structures of 22AG are the antiparallel and mixed parallel / antiparallel G-quadruplex DNA in Na^+^ buffer and K^+^ buffer, respectively.

### Synthesis of [(dmb)_2_Ru(obip)Ru(dmb)_2_][PF_6_]_3_.3CH_3_CN.H_2_O

A mixture of [Ru(dmb)_2_Cl_2_].2H_2_O (0.345 g, 0.60 mmol) and the bridging ligand obip (0.089 g, 0.30 mmol) were added to 20 mL ethylene glycol–water (9 : 1, v/v). The mixture was refluxed for 6 h under an argon atmosphere. The cooled reaction mixture was diluted with water (40 ml) and filtered to remove solid impurities. Ammonium hexafluorophosphate was added to the filtrate. The precipitated complex was dried, dissolved in a small amount of acetonitrile, and purified by chromatography over alumina, using MeCN–toluene (2:1, v/v) as eluent and dried in vacuo. Although single crystal of [(dmb)_2_Ru(obip)Ru(dmb)_2_]^4+^ has not yet been obtained, the red crystals of deprotonated [(dmb)_2_Ru(obip-H)Ru(dmb)_2_]^4+^ were grown from the mixture of acetonitrile-water (1:1) on standing in air at room temperature, yield: 271 mg, 50%. Calc. for C_72_H_69_F_18_N_16_OP_3_Ru_2_: C: 47.74; H: 3.84; N: 12.37. Found: C: 47.76; H: 3.85; N: 12.36. ESI-MS: m/z 691 (M-3PF6/2), 412 (M-4PF6/3). ^1^H NMR [(CD_3_) _2_SO]: δ (9.00 (1H,m), 8.70 (6H,m), 8.55 (1H,t), 8.50 (1H,s), 8.04 (2H,t), 7.92 (1H,s), 7.81 (2H,m), 7.74 (1H,d), 7.62 (4H,m), 7.40 (8H,m), 7.25 (1H,d), 7.19 (2H,m), 7.08 (2H,m), 6.91 (2H,m), 2.50 (24H,m). Crystal data for [(dmb)_2_Ru(obip-H)Ru(dmb)_2_]^4+^: C_72_H_69_F_18_N_16_OP_3_Ru_2_, *Mr* = 1811.48, triclinic, spacegroup *P-1*, *a* = 13.985(3) Å, *b* = 16.574(3) Å, *c*= 17.539(4) Å, α = 77.38(3), β = 79.07(3), γ = 83.44(3), *V* = 3883.9(13) Å^3^, *Z* = 2, *ρ*_calcd_ = 1.549 g cm^-3^, μ = 0.549 mm^-1^, *T* = 293 K, 14941 reflections collected, 7555 unique (*R*_int_ = 0.0687), R1 = 0.0754, wR2 = 0.1582 [*I*> 2σ*(I*)]*.*

### Circular Dichroism (CD) spectra

CD spectra were measured on a spectropolarimeter (Jasco J-810). The oligonucleotide samples were dissolved in three different solutions in this study: (a) 10 mM Tris-HCl in 100 mM Na^+^ buffer, (pH 7.4); (b) 10 mM Tris-HCl in 100 mM K^+^ buffer, (pH 7.4); (c) 10 mM Tris-HCl (pH 7.4); the corresponding samples of the DNA 22AG at a concentration of 10 μM were dissolved in different solutions and placed in a quartz cuvette. During the titration, aliquot (1-10 μL) of [(dmb)_2_Ru(obip)Ru(dmb)_2_]^4+^ solution was added to the cuvette, and the solutions were mixed by repeated inversion. After the solutions were mixed for ~5 minutes, the CD spectra were recorded. The titration processes were repeated until there was almost no change, indicating binding saturation had been achieved. For each sample, at least four spectrum scans were accumulated over the wavelength range of 200-350 nm at the temperature 25 °C in a 1.0 cm path length cell at a scanning rate of 50 nm /min. The instrument was flushed continuously with pure nitrogen throughout the experiment. The scan of the buffer alone was subtracted from the average scan for each sample.

### The stoichiometry interactions

The stoichiometry interactions between [(dmb)_2_Ru(obip)Ru(dmb)_2_]^4+^ and G-quadruplex DNA were obtained from the method of continuous variation analysis (Job plot). The concentrations of both metal complexes and DNA varied, while the sum of the concentrations of the two reactants was kept constant at 5 μM. In the solutions, the mole fraction of the Ru (II) complex varied from 1 to 0 in 0.05 increments. Each mixture was equilibrated at 5 °C for 12 h in the dark. The fluorescence intensities of these mixtures were measured on a Shimadzu RF-5000 at 25 °C using an excitation wavelength of 460 nm for [(dmb)_2_Ru(obip)Ru(dmb)_2_]^4+^. The *F*_max_ (fluorescence) was recorded in the range of 500–800 nm. Binding stoichiometry was obtained from the intercepts of the linear plot obtained by linear least-squares fits to the left- and right-hand portions of the Job plots.

### Thermal DNA denaturation experiments

Thermal DNA denaturation experiments were carried out with a PerkinElmer Lambda 850 spectrophotometer equipped with a Peltier temperature-control programmer (± 0.1) °C. Melting curves were collected by UV absorbance as a function of temperature. Absorbance changes at 295 nm vs temperature were collected at a heating rate of 1°C/min. The data were presented as (A - A_0_)/(A_f_ – A_0_) versus temperature, where A_f_, A_0_, and A are the final, the initial, and the observed absorbance at 295 or 260 nm, respectively.

### Quenching studies

Fluorescence quenching studies were carried out using the anionic quencher potassium ferrocyanide (K_4_Fe[CN)_6_]), monitoring the fluorescence intensity changes at 611 nm as a function of the quencher concentration. At least four measurements were taken and averaged. The data were plotted as I^o^/I versus quencher concentration [Q] according to the Stern–Volmer equation, as described earlier [34].

### Molecular docking studies

Both the antiparallel basket quadruplex (PDB ID 143D) and the mixed parallel/antiparallel structure (PDB ID 2E4I) (Waters were removed from the DNA PDB file) were used as an initial model to study the interaction between [(dmb)_2_Ru(obip)Ru(dmb)_2_]^4+^ and 22-mer telomeric G-quadruplex DNA. The structure of [(dmb)_2_Ru(obip)Ru(dmb)_2_]^4+^ was derived from the X-ray crystallographic data in CIF. When we prepare for Docking DNA and ligand ([(dmb)_2_Ru(obip)Ru(dmb)_2_]^4+^) PDB file (using Auto Dock Tools in AutoDock), necessary modifications are carried out including: (1) Add all hydrogens or just non-polar hydrogens; (2) Assign partial atomic charges to the ligand and the macromolecule (Gasteiger or Kollman United Atom charges); (3) Merge non-polar hydrogens and Set up rotatable bonds in the ligand; (4) output PDBQT files from traditional PDB files are also created for the side chain coordinates. Ligand docking was carried out with the AutoDock 4.2 Lamarckian Genetic Algorithm (LGA) [35,36]. In the autodocking, DNA was enclosed in the grid defined by Auto Grid having 0.375 Å spacing and parameters (supplied with the program package) were used for dispersion/repulsion, hydrogen bonding, electrostatics, and desolvation, respectively. Auto Grid performed a precalculated atomic affinity grid maps for each atom type in the ligand plus an electrostatics map and a separate desolvation map present in the substrate molecule. Then, during the AutoDock calculation, the energetics of a particular ligand configuration is evaluated using the values from the grids. The output from AutoDock was rendered with Accelrys Discovery Studio 3.0 Client.

### Competition dialysis

For each competition dialysis assay, 400 mL of dialysate solution containing 1µM [(dmb)_2_Ru(obip)Ru(dmb)_2_]^4+^ was placed into a beaker. A volume of 0.5 mL (at 10 µM monomeric unit) of each DNA samples was pipeted into a separate 0.5mL dialysis cassette (Pierce). The entire dialysis cassettes were then placed in the beaker containing the dialysate solution. The contents were allowed to equilibrate with continuous stirring for 24 h at room temperature. At the end of the equilibration period, DNA samples were carefully removed to microfuge tubes and taken to a final concentration of 1% (w/v) sodium dodecyl sulfate (SDS). The total concentration of [(dmb)_2_Ru(obip)Ru(dmb)_2_]^4+^ within each dialysis cassette was then determined spectrophotometrically using a wavelength of 460 nm. The free complex concentration (C_f_) was determined spectrophotometrically using an aliquot of the dialysate solution. The amount of bound [(dmb)_2_Ru(obip)Ru(dmb)_2_]^4+^ was determined by the difference between the total complex concentration and the free complex concentration (C_b_= C_t_-C_f_).

### TRAP (Telomeric repeat amplification protocol) assay

The ability of complex to inhibit telomerase in a cell-free system was assessed with a modification of the TRAP assay following previously published procedures [37]. Telomerase extract was prepared from MDA-MB-231 cells. The TRAP assay was performed in two steps: (a) telomerase-mediated extension of the forward primer (TS: 5′-AATCCGTCGAGCAGAGTT-3′) contained in a 20 μL reaction mixture comprising TRAP buffer (20 mM, pH 8.3), 68 mM KCl, 1.5 mM MgCl_2_, 1 mM EGTA, 0.05% v/v Tween-20, 0.05 μg of bovine serum albumin, 50 μM of each deoxynucleotide triphosphate, 0.1 μg of TS primer, and 3 μCi of [R-^32^P] dCTP. The protein (0.04 μg) was incubated with the reaction mixture ±agent (acid addition and quaternary dimethiodide salts) at final concentration of up to 50 μM for 20 min at 25 °C. Analysis buffer (no protein) control, heat-inactivated protein control, and 50% protein (0.02 μg) control were included in each assay. (b) While the mixture was being heated at 80 °C in a polymerase chain reaction (PCR) block of a thermal cycler for 5 min to inactivate telomerase activity, 0.1 μg of reverse CX primer (3′-AATCCCATTCCCATTCCCATTCCC-5′) and 2 units of Taq DNA polymerase were added. A three-step PCR was then performed: 94 °C for 30 s, 50 °C for 30 s, and 72 °C for 1 min for 31 cycles. Telomerase-extended PCR products in the presence or absence of compounds were determined by electrophoretic separation using 8% w/w acrylamide denaturing gels. ELISA experiment was performed according to the Telo TAGGG Telomerase PCR ELISA Kit (Roche, #11854666910). Briefly, Hela cells treated or untreated with [(dmb)_2_Ru(obip)Ru(dmb)_2_]^4+^ at ^MTT^IC_50_ = 120uM were both extracted protein and adjusted to a final concentration of 1 μg/μl. The following TRAP reaction and hybridization as well as ELISA procedure were strictly based on the kit guidance. The results were expressed as the absorbance of the samples at 450 nm with a reference wavelength of approx 690 nm.

### MTT assay for cell proliferation determination

Cell proliferation was analyzed by MTT assay as described previously [38]. Briefly, after the starting cells (5 ×10^3^/well) were treated by [(dmb)_2_Ru(obip)Ru(dmb)_2_]^4+^ or cisplatin for 24 h, 48 h and 72 h in a 96-well plate with untreated cells as the control, MTT (5 mg/ml) was added into each well at 10% (V/V). The MTT assay was quantified at 570 nm with a chemi-luminescent microplate reader (SpectraMAX 250, Gene Co. Ltd.) after labeling for 3 h. Inhibition effect was calculated by the value of OD_570_ of the untreated cells to subtract that from the treated cells then divided by that of the untreated cells. The median inhibition concentration (^MTT^IC_50_), which means the complex concentration when the cells were inhibited by 50%, was obtained according to the linear plots collected via MTT assay. In our experiment, we used two cancer cell lines of human cervical cancer Hela cells (RR-B51S, ATCC# PTA-5258) and chronic myelogenous leukemia cell line of K-562 (ATCC# CCL-243). To evaluate their might side effects to human normal cells, we choose human normal fibroblast cell line (BJ, ATCC# CRL-2522) to perform a parallel experiment. To compare the treatment effects with other clinical used chemotherapy drugs, the widely used cisplatin was chosen as the reference compound. To better mimic the clinical chemotherapy, we used clinically relevant concentration gradient of 0, 6.25uM. 12.5uM, 25uM and 50 uM during our MTT experiment.

### TUNEL (TdT-mediated dUTP nick end labelling) Assay

Apoptosis was usually used to evaluate an anti-cancer drug to exclude the possibly induced necrosis during treatment. TUNEL assay was chosen to detect the DNA strand breaks during apoptosis instead of necrosis in this experiment. In principle, during apoptosis, DNA strand breaks can be labelled at the exposed free 3’-OH termini with modified nucleotides in an enzymatic reaction by terminal deoxynucleotidyltransferase (TdT).The fluorescein labels incorporated in nucleotide polymers can be detected and quantified by fluorescence microscopy or flow cytometry. The protocol followed the kit of In Situ Cell Death Detection, Fluorescein (Roche, #11684795910). Briefly, Hela cells treated or untreated with [(dmb)_2_Ru(obip)Ru(dmb)_2_]^4+^ at ^MTT^IC_50_=120 uM for 24, 48, 72 h respectively at 37 °C or DNase I recombinant (3000 U/mL-3 U/mL in 50 mM Tris-HCl, PH 7.5, 1 mg/ml BSA) for 10 min at 15-25 °C were fixed and labelled. The sequential labelling protocol strictly followed the kit instructions. The labelled samples were directly analysed under a fluorescence microscope using an exciting wavelength in the range of 450-500 nm (488 was taken in this experiment) and detection in the range of 515-565. Quantification of the green fluorescence positive cells was performed by taking average of at least five view fields.

### Statistics process

All of the experiments were performed in triplicate if not specified otherwise. The results were expressed in mean ± one standard deviation (S.D.). Statistical signiﬁcance between and among groups was determined by Student’s paired two-tailed t test or one-way analysis of variance, using Dunnett’s multiple comparison tests as the post hoc analysis. Ap < 0.05 was considered signiﬁcant difference (GraphPad Prism Two Way RM ANOVA Bonferroni Posttests).

## Results and Discussion

### Synthesis and characterization

**Figure 2:**
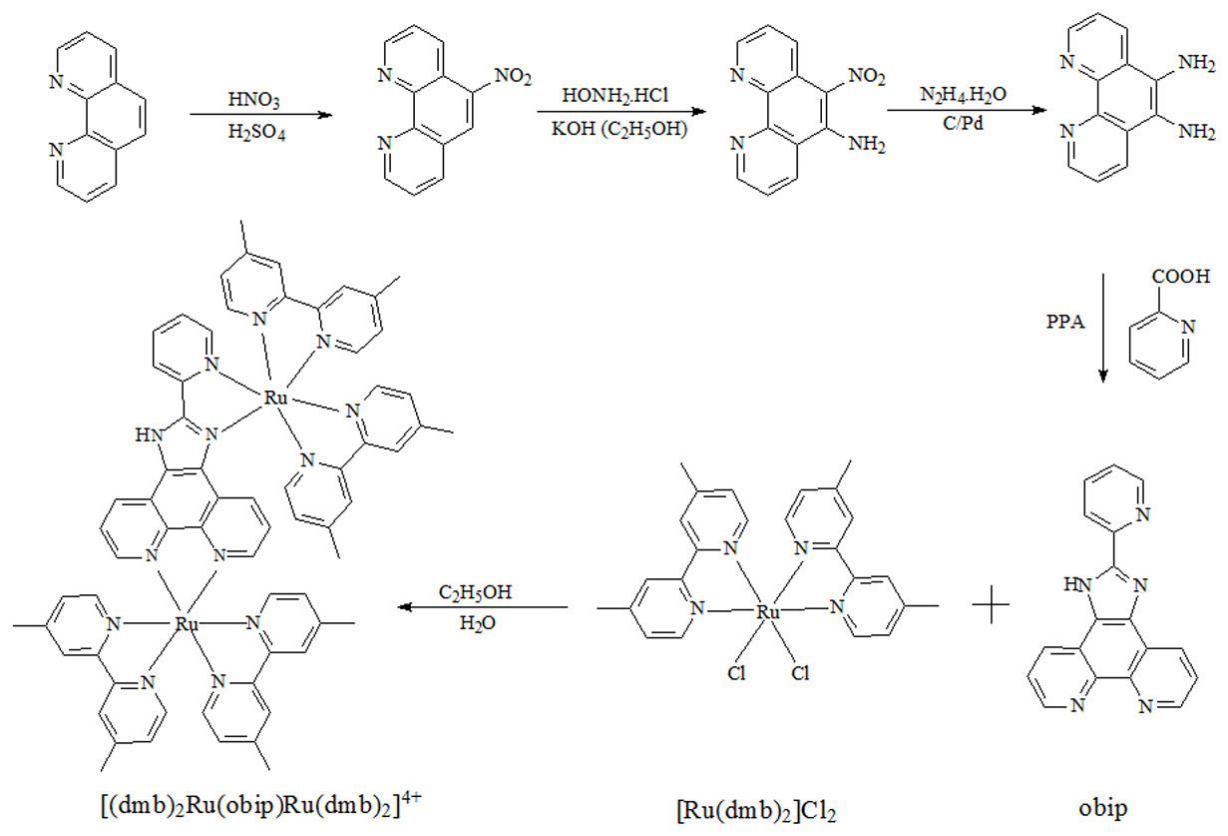
Synthetic routes for the preparation of the complex [(dmb)_2_Ru(obip)Ru(dmb)_2_]^4+^.

An outline of the synthesis of the ligand and complex is presented in Figure 2. The obip is an asymmetric ligand which can coordinate to metal ions via two different sites, one is the nitrogen atoms of 1,10-phenanthroline and the other is composed of one of the pyridine ring and one of the imidazole ring. Indeed, treatment of 1 equivalent of obip ligand with 2 equivalents of [Ru(dmb)_2_Cl_2_].2H_2_O provided the new asymmetric dinuclear ruthenium(II) complex in high yield. Crystals were grown by slow diffusion of acetonitrile into a water solution of the complex in a thin, long tube. The crystal structure of deprotonated [(dmb)_2_Ru(obip-H)Ru(dmb)_2_]^4+^ was solved by direct methods and refined using full-matrix least-squares/difference fourier techniques using SHELXTL. The ORTEP diagram of the cation is shown in Figure 1D. The complex consists of a deprotonated [(dmb)_2_Ru(obip-H)Ru(dmb)_2_]^3+^ cation, three disordered PF_6_^-^ anions, three CH_3_CN and one H_2_O solvent molecule. All the pyridyl rings from the dmb and obip ligands are essentially planar. The geometry about two Ru centers is a distorted octahedral, with angles in the 78.4–102.7 °and 169.7–173.6 ° ranges. The Ru-N pyridine distances vary in the 2.051–2.084 Å ranges, which are consistent with the typical values observed for related Ru^II^ polypyridyl complexes [39,40]. However, the Ru–N imidazo distance (2.136 Å) is significantly larger than the Ru-N pyridine distances. The inequivalence of the two Ru–N bonds indicates that the pyridine group has stronger coordination ability than the imidazole. 

### CD titration

**Figure 3:**
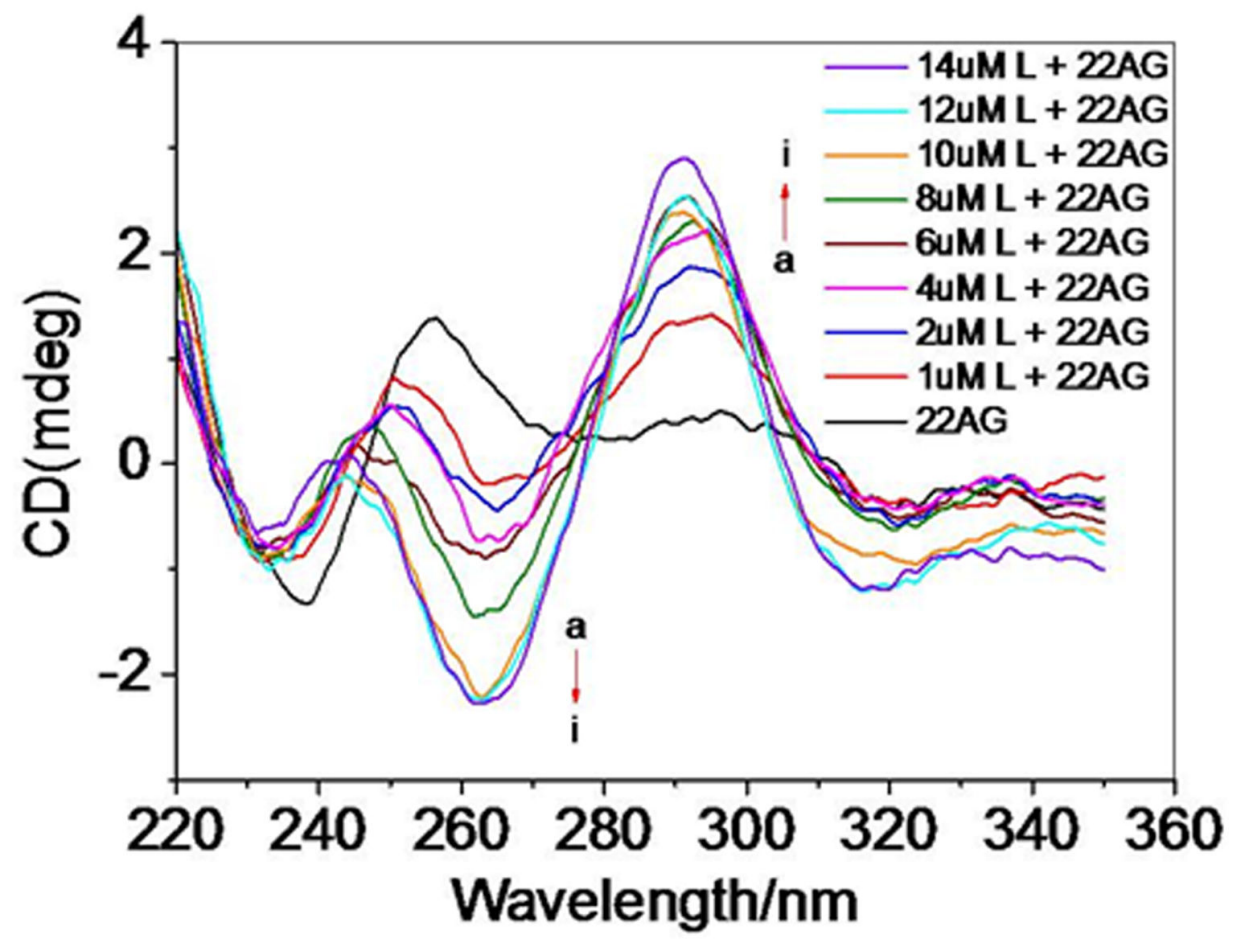
CD titration of 22AG with complex in 10 mM Tris buffer. CD titration spectra of 22AG 20 µM in 10 mM Tris-HCl: with (a) 0 µM, (b) 1µM, (c) 2 µM, (d) 4 µM, (e) 6 µM, (f) 8 µM, (g) 10 µM, (h) 12 µM, (i) 14 µM [(dmb)_2_Ru(obip)Ru(dmb)_2_]^4+^.

CD has been used to study 3D-structures, ligand binding, effect of cations, and the kinetics of quadruplex formation [41,42]. The parallel quadruplexes are characterized by a dominant positive band at 260 nm, whereas the spectra of antiparallel forms display a negative band at this wavelength and a positive band at 295nm. In addition, all quadruplexes display another positive band around 215 nm [43,44]. [(dmb)_2_Ru(obip)Ru(dmb)_2_]^4+^ induced formation of the human telomeric intramolecular G-quadruplex structure in the absence of K^+^ or Na^+^ was shown in Figure 3. Without any metal cations, the CD spectra of the human telomeric 22AG at room temperature exhibited a negative band centered at 235 nm, a major positive band at 257 nm, which probably corresponded to the signal of the random-coil 22AG (characterized by a positive peak at 257 nm) [45]. However, upon addition of [(dmb)_2_Ru(obip)Ru(dmb)_2_]^4+^ to the 22AG oligonucleotide, a dramatic variance in the CD spectrum was observed. The maximum at 257 nm was gradually suppressed and shifted to 245 nm, while the band centered at about 290 nm increased dramatically with increasing of [(dmb)_2_Ru(obip)Ru(dmb)_2_]^4+^ concentration. Meanwhile, a major negative band at about 260 nm started to appear and increased sharply. Approximately 15 min after the addition of complex (10 µM), the new peaks and troughs ceased to increase in elliptic intensity, and the reaction probably reached, or was very close to, the equilibrium state, the CD spectrum of this DNA conformation was virtually identical to the CD spectra of basket antiparallel intramolecular G-quadruplexes described in previous studies, where the major positive band was usually observed around 290 nm with a negative band at 265 nm and a smaller positive band at 246 nm [43-45]. Our data show that complex [(dmb)_2_Ru(obip)Ru(dmb)_2_]^4+^ shows prominent ability to promote the assembly of intramolecular G-quadruplexes from the random coil 22AG. However upon addition of [(dmb)_2_Ru(obip)Ru(dmb)_2_]^4+^ to the 22AG in Na^+^ or K^+^ buffer, no obvious spectral changes were observed (Figure s1.), which implied that the conformation of G-quadruplex was stabilized by Na^+^ or K^+^, [(dmb)_2_Ru(obip)Ru(dmb)_2_]^4+^ could not disturb the conformation of G-quadruplex at high ionic strength.

### UV melting study

**Figure 4:**
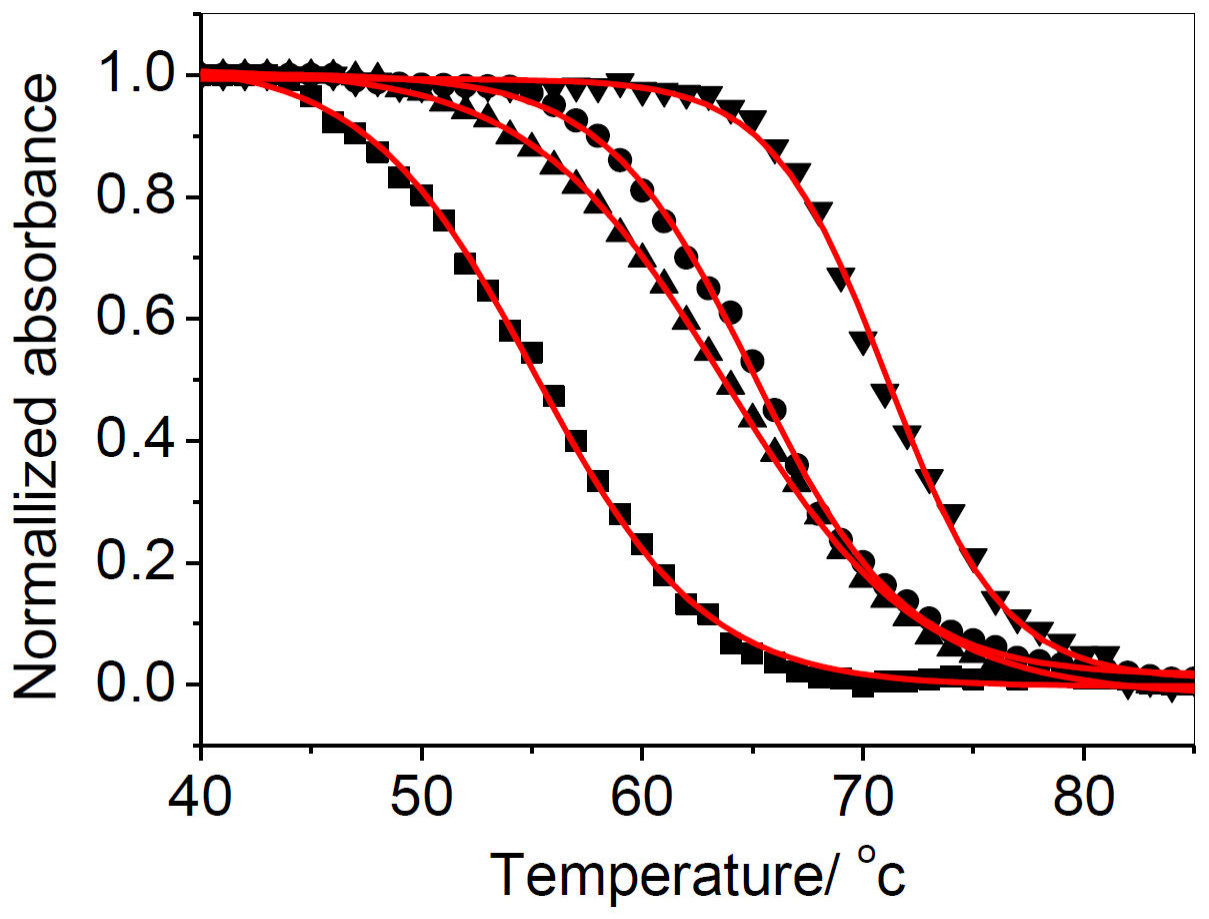
Melting of the G-quadruplex assessed by UV absorbance at 295 nm. Normalized UV melting curves for 10 µM G-quadruplex (■), 10 µM G-quadruplex + 10 µM [(dmb)_2_Ru(obip)Ru(dmb)_2_]^4+^ (●)in a buffer of 100 mM NaCl, 10 mMNaH_2_PO_4_/Na_2_HPO_4_, 1mM Na_2_EDTA; 10 µM G-quadruplex (▼) 10 µM G-quadruplex + 10 µM [(dmb)_2_Ru(obip)Ru(dmb)_2_]^4+^ (▲)in a buffer of 100 mM KCl10 mM K_2_HPO_4_/K_2_HPO_4_, 1mM K_2_EDTA (pH 7.0).

To gain further insights into the interaction between [(dmb)_2_Ru(obip)Ru(dmb)_2_]^4+^ and G-quadruplex, we examined the ability of [(dmb)_2_Ru(obip)Ru(dmb)_2_]^4+^ to stabilize G-quadruplex DNA by thermal denaturation profiles. Consistent with the previous studies [46-52], 295 nm was chosen to study the complex influences on the stability of G-quadruplex. As expected, the melting profiles of 22AG in the absence of K^+^ or Na^+^ showed almost no transition (not shown here), suggesting that it did not form a stable G-quadruplex structure. In terms of dissociation of G-quadruplex, the T_m_ of the monovalent ion K^+^ induced intramolecular G-quartet structure of 22AG sequence was higher than its Na^+^ counterpart. With the ratio of [(dmb)_2_Ru(obip)Ru(dmb)_2_]^4+^ to 22AG equaled 1, the transition temperature of the G-quadruplex increased from 55.1 to 63.7 °C in Na^+^ buffer and increased from 65.0 to 71.1°C in K^+^ buffer (Figure 4). It was well known that an increase in the melting temperature of the quadruplex indicated better stabilizing effect. The results implied that [(dmb)_2_Ru(obip)Ru(dmb)_2_]^4+^ strongly stabilized the G-quadruplex in Na^+^ or K^+^ buffers. As it was reported, telomerase inhibition activity of drugs closely correlates with their stabilization ability of quadruplex structure. Therefore, [(dmb)_2_Ru(obip)Ru(dmb)_2_]^4+^ might be a promising candidate for potent telomerase inhibiter and anticancer drug.

### Binding stoichiometry and fluorescence quenching

Binding stoichiometry with G-quadruplex was investigated through luminescence based Job plot. One major inflection point for [(dmb)_2_Ru(obip)Ru(dmb)_2_]^4+^ at about x = 0.50 was observed. The data were consistent with a 1:1 [quadruplex]/ [complex] binding mode (Figure s2). 

**Figure 5:**
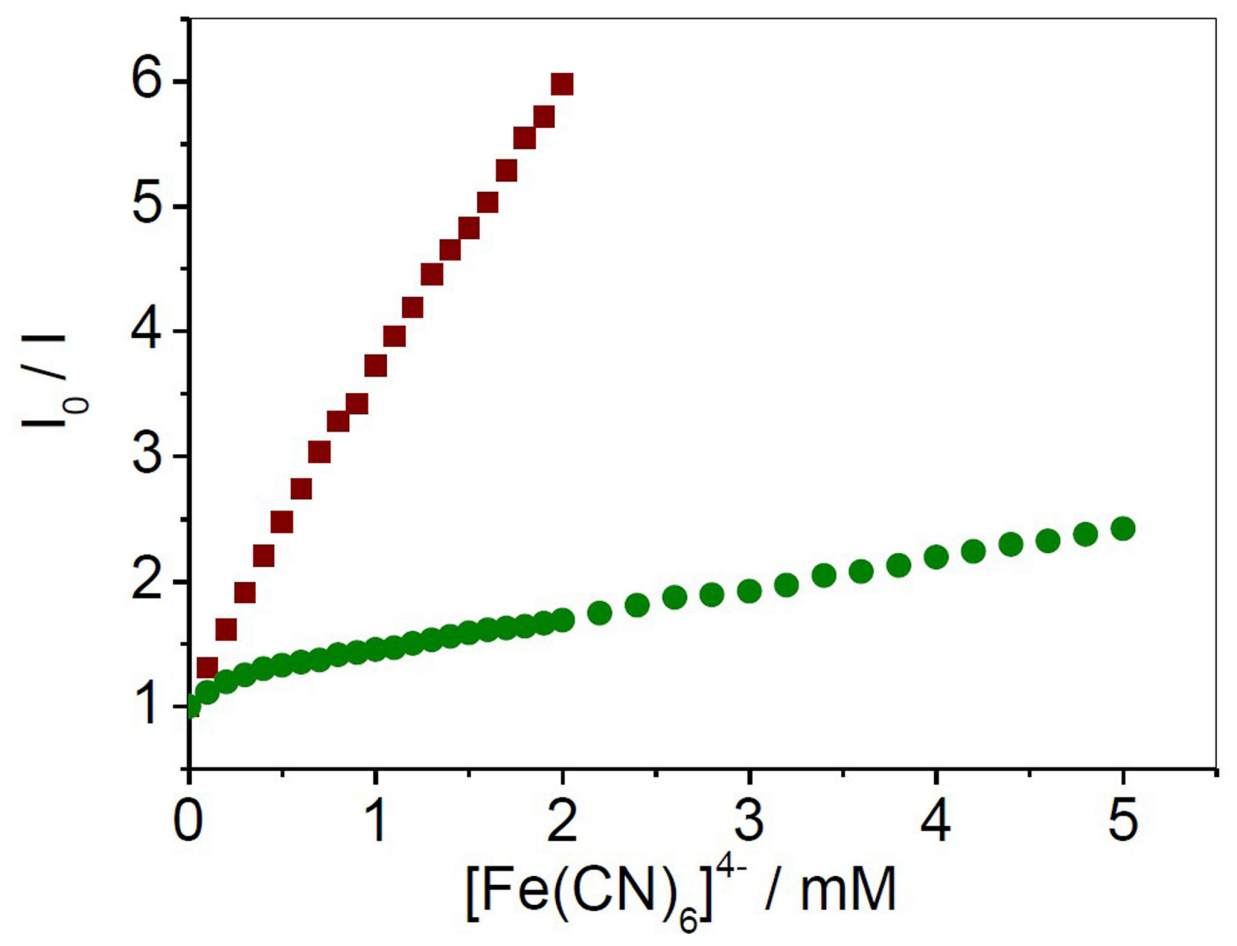
Binding modes assessed by Emission quenching studies. Emission quenching curves of [(dmb)_2_Ru(obip)Ru(dmb)_2_]^4+^ with increasing concentration of quencher [Fe(CN)_6_]^4-^ in the absence: (■) and presence of G-quadruplex DNA (•), [Ru] = 4μM, [DNA]/[Ru] = 1.

Small molecules can potentially bind to G-quadruplex by externally stacking below the quartets, intercalating between the quartets, or nonspecifically binding to some random location on the DNA strand [53,54]. The molecules bound to the surface of the helix or quartets will be accessible to the quencher while those buried by intercalation inside the helix or quartets will be protected from the quencher [55,56]. To elucidate the G-quadruplexes binding mode(s) of [(dmb)_2_Ru(obip)Ru(dmb)_2_]^4+^, the fluorescence quenching experiments was performed. Ferrocyanide ion proved to be an excellent quencher for the complex in the presence of DNA. The [Fe(CN)_6_]^4-^ ion will be prevented from entering into the quartets due to the electrostatic barrier from the phosphate group and consequently very little quenching will be observed in the case of true intercalators, thus giving quenching constants of nearly zero. In appropriate buffer and at a ratio of 1:1 (ruthenium to nucleotide) the emission was monitored at the emission maxima of complex at various concentrations of ferrocyanide, at 20 °C. In the absence of DNA, sufficiently low quencher concentrations were utilized to yield linear dependences on quencher concentrations; a value of *K*_sv_ was derived to be 2.5*10^3^ M^-1^ (Figure 5). On addition of G-quadruplex DNA, the slope of the Stern-Volmer plot declined drastically, giving a much less *K*_sv_ value of 300 M^-1^, but the fluorescence of complexes was quenched by [Fe(CN)_6_]^4-^ under high quencher concentrations, which implies that intercalation of [(dmb)_2_Ru(obip)Ru(dmb)_2_]^4+^ between successive G-tetrads would seem to be an unlikely binding mode.

### Molecular docking studies

**Figure 6:**
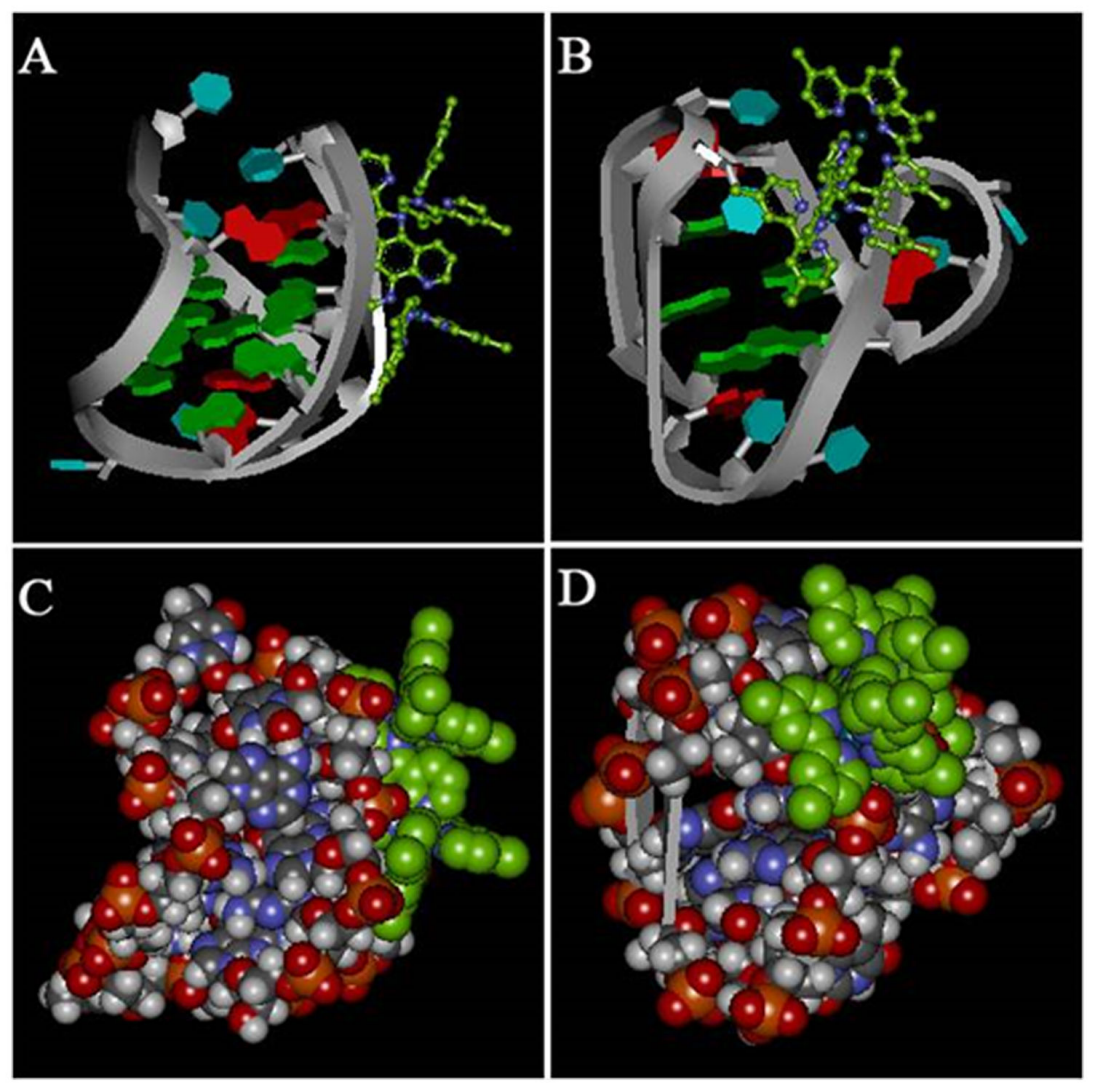
Binding modes assessed by molecular docking studies. A) The schematic diagram of the interaction of [(dmb)_2_Ru(obip)Ru(dmb)_2_]^4+^ with the antiparallel G-quadruplex structure, B) the mixed parallel/antiparallel G-quadruplex structure. G in green yellow, A in red, T in cyan and [(dmb)_2_Ru(obip)Ru(dmb)_2_]^4+^ in green and blue, C) the complex, the antiparallel G-quadruplex and D) the mixed parallel/antiparallel G-quadruplexare shown in CPK mode.

Quadruplex DNA structure polymorphism leads to binding mode uncertainties between the compounds and their targeted quadruplex, making the rational designs of G4-directed small molecules difficult [57,58]. Molecular docking studies were carried out to evaluate the binding modes between G-quadruplex DNA and [(dmb)_2_Ru(obip)Ru(dmb)_2_]^4+^. Crystal structure analysis shows that [(dmb)_2_Ru(obip)Ru(dmb)_2_]^4+^ has a large planar aromatic area (obip) and two metal cations, which possess the potential to fit the geometric structure of G-quadruplex. The docking study confirms that each intramolecular G-quadruplex molecule binds one [(dmb)_2_Ru(obip)Ru(dmb)_2_]^4+^ molecule (Figure 6). It was also found that the binding modes between the complex and G-quadruplex DNA via a combination manner: electrostatic/metal-phosphate interactions (between the metal and DNA’s backbone), π-π end-stacking (between the ligand and the G-quartet) and hydrophobic interactions. Both obip and dmb ligands prefer to stack in the center of a terminal G-quartet end. Two metals not only provides a structural locus to restrict the geometry of the aromatic core but also strengthens its binding affinities with the G-quadruplex DNA [59]. Considering the planar aromatic structure of the ligands (both obip and dmb), possible binding of the hydrophobic interaction in the grooves of the G-quadruplexes DNA should also be considered as an alternative or additional possibility to the end stacking mode. The modeling study reveals that complex binds to both G-quadruplexes with a calculated binding energy of ^~^-10 kcal/mol (lower than binding energy of duplex DNA ^~^ -5 kcal/mol) (Figure s3.), similar to end-stacking of previously reported G-quadruplex binders [60]. Thus, molecular modeling studies implied that the title complex preferentially bind G-quadruplex over duplex DNA and confirmed the excellent complementarity in binding modes. 

### Competition Dialysis

**Figure 7:**
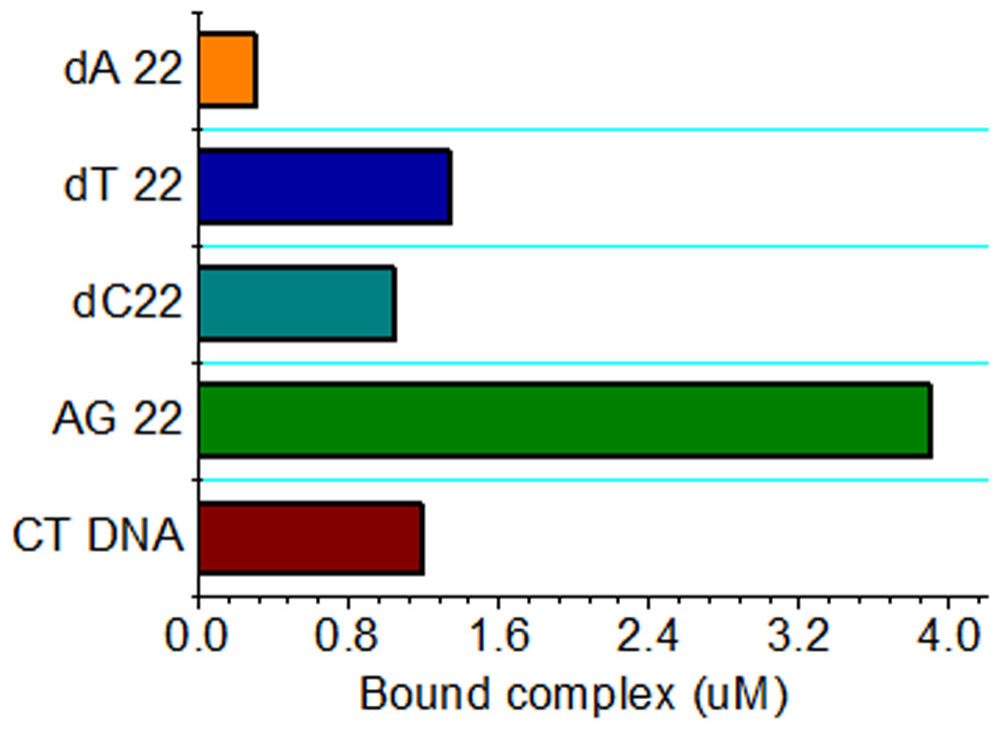
Selectivity for G-quadruplex DNA assessed by competition dialysis. Results of competition dialysis experiment with the amount of [(dmb)_2_Ru(obip)Ru(dmb)_2_]^4+^ bound to each DNA structure plotted as a bar graph.

A high selectivity for G-quadruplex DNA over other forms of DNA and low cytotoxicity are essential properties for any G-quadruplex interacting compounds when considered to be used as telomerase inhibitors or probes for *in vivo* detection of G-quadruplex DNA. Multiple DNA competition dialysis is a useful method, recently developed by the Chaires’ laboratory to screen compounds with excellent sequence and structure specificity within a broad panel of DNAs [61-63]. Among the DNA used in the present study, 22AG could form the G-quadruplex structure, dC22, dT22 and dA22 were single-strand pyridine and purine structures, respectively, and calf thymus DNA was a native duplex DNA structure. More products accumulated in the dialysis cassette containing the structural form with the highest complex binding affinity. Competition dialysis results for [(dmb)_2_Ru(obip)Ru(dmb)_2_]^4+^ were shown as a bar graph in Figure 7. A strong interaction of [(dmb)_2_Ru(obip)Ru(dmb)_2_]^4+^ with G-quadruplexes structures and weak interaction with all single-stranded and duplex DNAs are easily visualized from the data of this assay (Figure 7). Competition dialysis results further support the above proposal that the title complex preferentially bind G-quadruplex over duplex DNA. 

### TRAP Assay and MTT Assay

**Figure 8:**
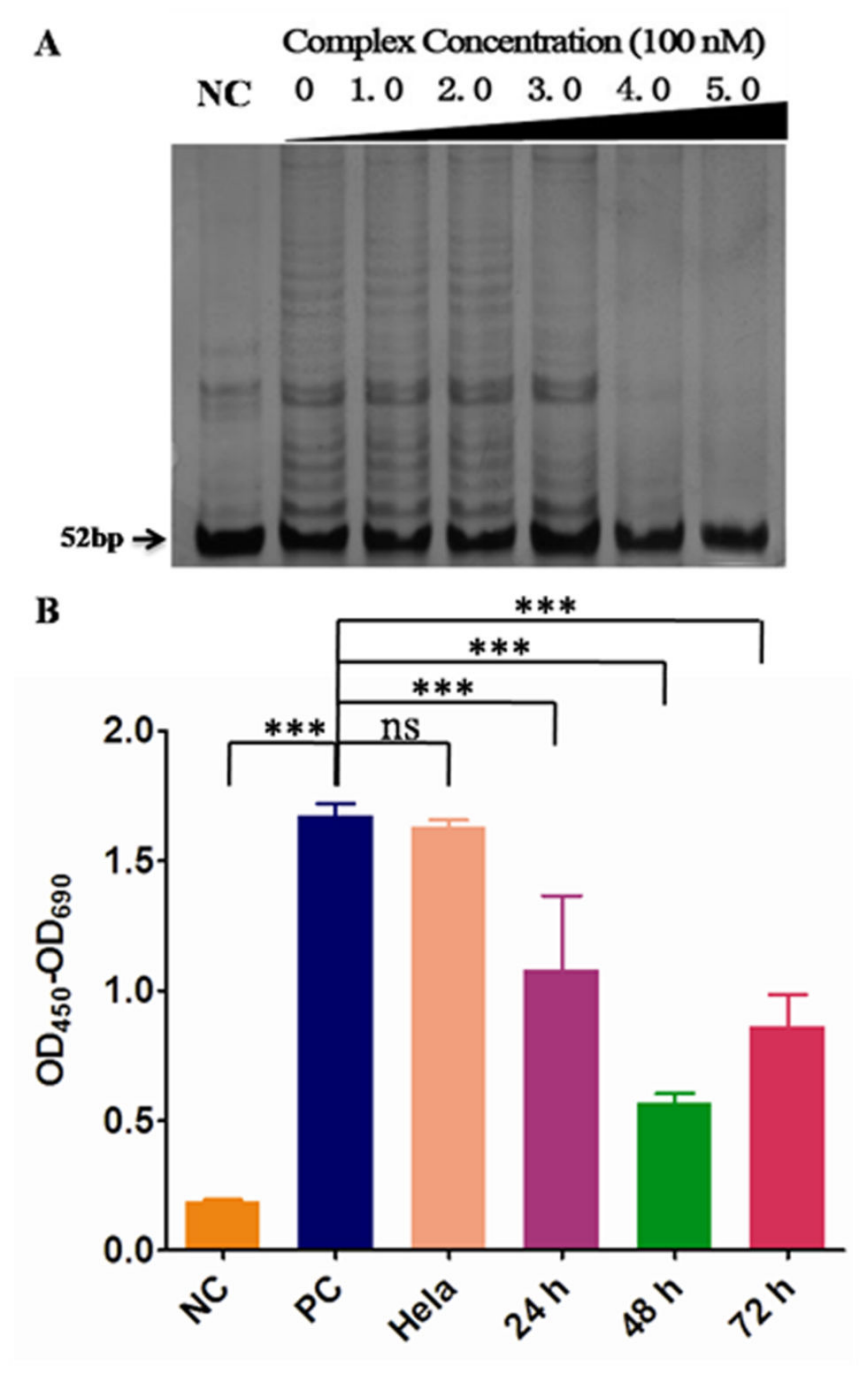
Telomerase inhibition by [(dmb)_2_Ru(obip)Ru(dmb)_2_]^4+^. TRAP Assay with Hela cells at gradient concentrations of [(dmb)_2_Ru(obip)Ru(dmb)_2_]^4+^ for 24 h shown in (A), quantitative analysis of the telomerase inhibition by ELISA shown in (B). PC means positive control with HEK293 provided by the kit, NC means the negative control obtained by heating Hela cell protein for 10 min at 85 °C. Hela means Hela cells untreated with [(dmb)_2_Ru(obip)Ru(dmb)_2_]^4+^. 24 h, 48 h, 72 h means Hela cells respectively treated with the complex at ^MTT^IC_50_= 120 nM for 24 h, 48 h and 72 h. Data were represented as mean +/- S.E.M. * means significant difference by GraphPad Prism5 One way ANOVA (Tukey's Multiple Comparison Test).

**Figure 9:**
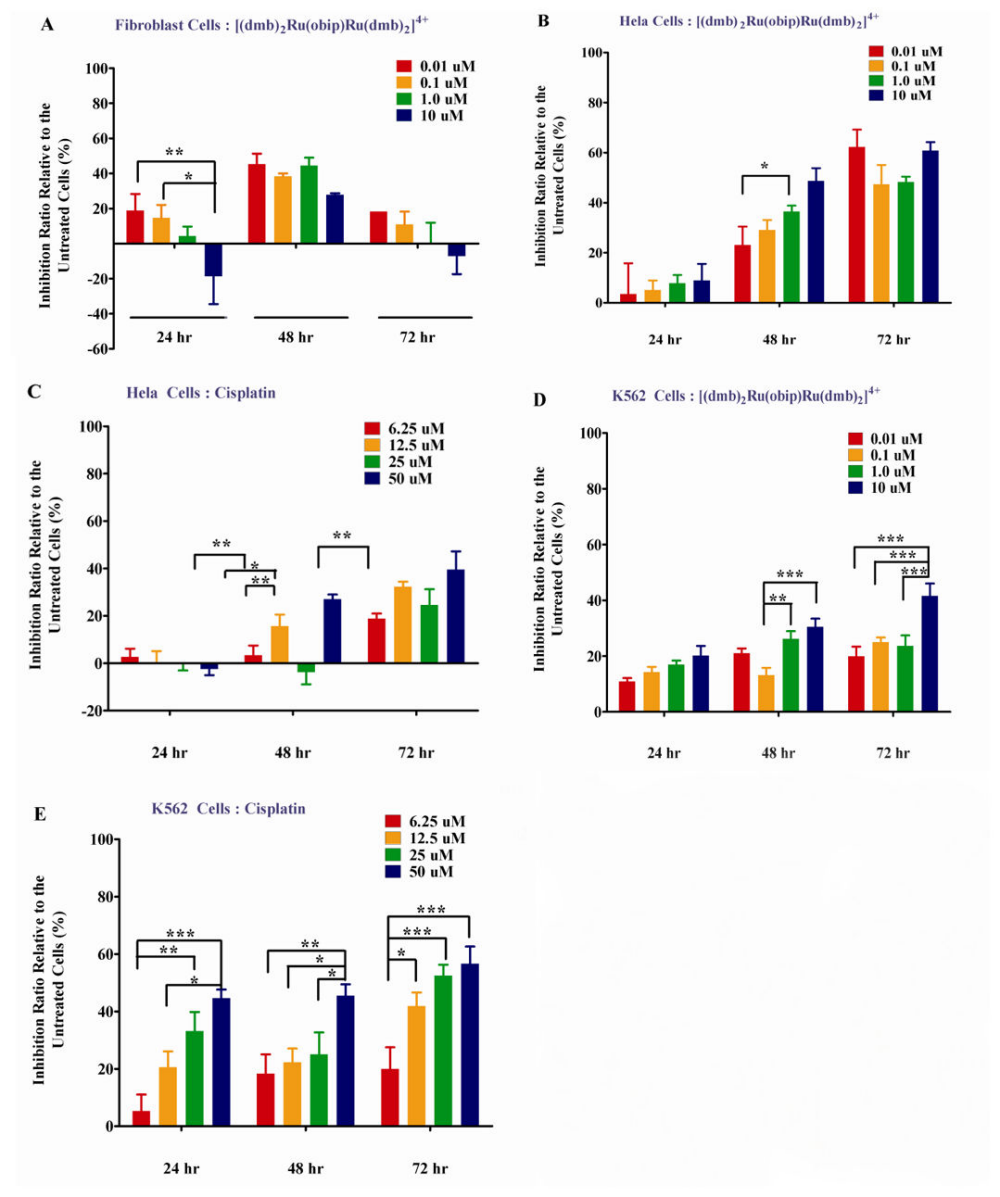
Cytotoxic effects of complexes on cells. MTT assay was performed on human normal fibroblast cells treated with [(dmb)_2_Ru(obip)Ru(dmb)_2_]^4^ A), human cervical cancer Hela cells treated with [(dmb)_2_Ru(obip)Ru(dmb)_2_]^4+^ at gradient concentrations B), Hela cells treated with a clinical chemotherapeutic cisplatin C), K562 cells treated with [(dmb)_2_Ru(obip)Ru(dmb)_2_]^4^ D) and K562 cells treated with cisplatin respectively at gradient concentrations. The inhibition ratio was collected as the average of triplicate wells relative to that of the untreated wells. Result shown here was the representative one among three independent experiments. Data were presented as mean +/- S.E.M.* means P < 0.05, ** means P < 0.005, *** means P < 0.0001 significant difference was observed by GraphPad Prism5 Two-way RM ANOVA (Bonferroni posttests).

These encouraging aforementioned results prompted us to investigate whether [(dmb)_2_Ru(obip)Ru(dmb)_2_]^4+^ would actually inhibit telomerase activity at the cellular level by TRAP assay (Figure 8) and further inhibit cancer cell proliferation by MTT assay (Figure 9). TRAP assay has been widely used to detect the telomerase inhibition in both qualitative and quantitative manners [36,37]. As shown in Figure 8, the title complex showed high activity with ^tel^IC_50_ (the complex concentration at which the telomerase activity was inhibited by 50%) equal to 350 nM (^tel^IC_50_), which was consistent with the marked increase in T_m_ found for [(dmb)_2_Ru(obip)Ru(dmb)_2_]^4+^ in thermal denaturing studies. It displays nearly 100% inhibition of telomerase activities at the concentration of around 400 nM. To quantitatively estimate the telomerase inhibition by the title complex at ^MTT^IC_50_ (the complex concentration at which the cell proliferation was inhibited by 50%), ELISA experiment was carried out (Figure 8B). The telomerase activity of human cervical cancer cell line of Hela cells was significantly (P<0.0001) inhibited by [(dmb)_2_Ru(obip)Ru(dmb)_2_]^4+^ at the ^MTT^IC_50_ (120 nM) after 24 h, 48 h and 72 h treatment compared with either the negative control (NC) or the positive control (PC). There is no significant (ns, P>0.05) difference between PC and Hela cells. The inhibitory effect of [(dmb)_2_Ru(obip)Ru(dmb)_2_]^4+^ emerged after 24 h treatment and culminated around 48h treatment. Numerous small molecules were optimized to improve their selective interaction with human telomere derived intramolecular G-quadruplex instead of duplex DNA. The first generation of G-quadruplex-interacting compounds such as anthraquinones, cationic porphyrins, fluorenones, and acridines have high ^tel^IC_50_ values in the micromolar range but with lower selectivity for G-quadruplexes. Current developments brought some potent G-quadruplex interacting compounds with relatively lower ^tel^IC_50_ values (100–800 nM) by a modified TRAP assay meanwhile with lower cytotoxicity [63-66]. Up to now, the most potent G-quadruplex-interacting compound reported is telomestatin, a natural product, with only 5 nM of ^tel^IC_50_ value. Neidle, Che and co-workers have described some metalporphyrin complexes and planar Pt complexes that are telomerase inhibitors, with activities comparable to those of free porphyrin ligands [63-65]. Indeed, this ^tel^IC_50_ value of [(dmb)_2_Ru(obip)Ru(dmb)_2_]^4+^ is comparable to those of some of the most potent G-quadruplex interacting compounds reported in the literature [acridine derivatives, ethidium derivatives, quinolines, Ni complexes, Pt complexes (^tel^IC_50_ of ca.100-800nM)] and lower than those of the first generation of G-quadruplex-interacting compounds such as anthraquinones, cationic porphyrins, fluorenones, and acridines (^tel^IC_50_ values in the micromolar range) [5-9,20-23,63-65]. 

To detect whether this inhibition of telomerase activity really led to the inhibition of cancer cells proliferation meanwhile without significant side-effects to the human normal cells, in vitro cytotoxicity assay of [(dmb)_2_Ru(obip)Ru(dmb)_2_]^4+^ was evaluated by means of MTT assay (using cisplatin as the positive control) against human normal fibroblast cells and two human cancer cells of Hela and K562 at a gradient concentration. The results indicated that the cancer cells tested are susceptible to the complexes. As shown in Figure 9, the proliferation of Hela cancer cells was heavily inhibited by [(dmb)_2_Ru(obip)Ru(dmb)_2_]^4+^ with time lapse, while that of the human normal fibroblast cells was not affected much (with maximum inhibition ratio of 45.2%). The complex inhibited both cancer cells proliferation in a time dependent manner (P<0.0001) (Table s1). Significant inhibition was also found with K562 cells when treated with cisplatin. These results indicated that the complex [(dmb)_2_Ru(obip)Ru(dmb)_2_]^4+^ held great promise to be a good drug candidate for anticancer therapy in the future clinical application with much lower effective dosage. It was also worth noting that [(dmb)_2_Ru(obip)Ru(dmb)_2_]^4+^ showed a distinct preference for HeLa cells, thus HeLa cells were chosen as a cell model for further investigation of the mechanisms underlying the antiproliferative action of [(dmb)_2_Ru(obip)Ru(dmb)_2_]^4+^.

### TUNEL assay

**Figure 10:**
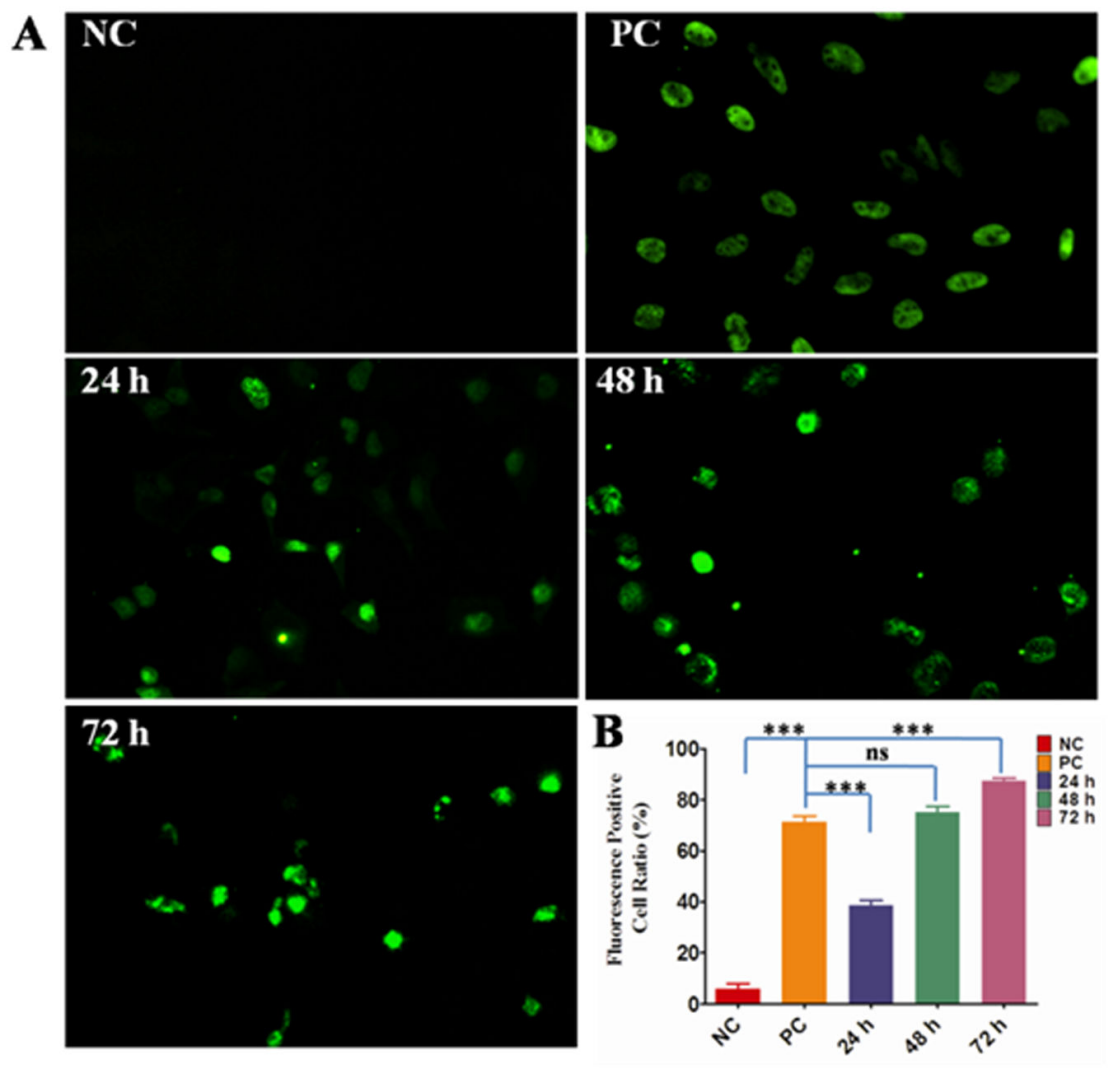
TUNEL assay with Hela cells to detect the oligonucleosomal DNA cleavage during cell apoptosis induced by [(dmb)_2_Ru(obip)Ru(dmb)_2_]^4+^. A) The representative green fluorescence images taken under microscope after treatment with [(dmb)_2_Ru(obip)Ru(dmb)_2_]^4+^ at ^MTT^IC_50_ = 120 nM at different time points, B) the percentage of green fluorescence positive cells after [(dmb)_2_Ru(obip)Ru(dmb)_2_]^4+^ treatment in comparison with both the negative and positive controls. NC means the untreated Hela cells used as the negative control, PC means Hela cells treated with DNase I recombinant (3000U/ml-3U/ml in 50 mM Tris-HCl, PH 7.5, 1 mg/ml BSA) for 10 min at 15-25 °C used as the positive control. 24 h, 48 h and 72 h mean Hela cells treated with [(dmb)_2_Ru(obip)Ru(dmb)_2_]^4+^ for 24 h, 48 h and 72 h respectively before labelling. Scale bar 25 um. Data were presented as mean +/- S.E.M. * means significant difference was observed by GraphPad Prism5 One way ANOVA (Tukey's Multiple Comparison Test).

Since this complex [(dmb)_2_Ru(obip)Ru(dmb)_2_]^4+^ is potential to be developed as a cancer therapy drug, it is essential to determine whether this inhibition of telomerase activity and proliferation of cancer cells will further induce apoptotic death. Therefore, TUNEL assay was performed and the results were shown in Figure 10. Treatment with this complex at the concentration of ^MTT^IC_50_ at various time points showed that Hela cells were induced to undergo apoptosis indicated by the green fluorescence. With time lapse, the percentage of green fluorescence positive cells significantly (P<0.0001) increased (from 38.4% at 24 h to 87.4% at 72 h), indicating that DNA strand breaks, accumulate over time and eventually led to apoptosis. After 48 h treatment, the percentage of the green fluorescence positive cells treated by the title complex had already been comparable (P>0.05) with the positive control (PC) treated by DNaseI recombinant (74.9% of 48 h *vs* 71.3% of PC). On the other side, after 72 h incubation, Hela treated cells had a significantly (P<0.0001) higher green fluorescence positive cells than the positive control cells. These data demonstrated that the title complex was a potent apoptotic inducer. The mechanism through which G-quadruplex promoted by complexes and induced tumor cell apoptosis has been demonstrated [29-32]. It was shown that a variety of telomeric G-tail oligodeoxynucleotides (TG-ODNs) exhibited anti-proliferative activity against many tumor cells in culture. Systematic mutational analysis of the TG-ODNs suggests that the antiproliferative activity depends on the G-quadruplex conformation of these TG-ODNs. TG-ODNs were also shown to induce poly (ADP-ribose) polymerase-1 cleavage, phosphatidylserine flipping, and caspase activation, indicative of induction of apoptosis. TG-ODN–induced apoptosis was largely ataxia telangiectasia mutated (ATM) dependent. It was suggested that G-quadruplex inhibited tumor cell proliferation and induced apoptosis by activating the JNK pathway in an ATM-dependent manner as evidenced by elevated phosphorylation of JNK and c-Jun. These reported results combined with our TUNEL data propose/hint that the mechanism underlying the anti-proliferation and apoptosis induced by [(dmb)_2_Ru(obip)Ru(dmb)_2_]^4+^ may share the similar cellular signaling pathway. This will shed light on our further study to clarify how [(dmb)_2_Ru(obip)Ru(dmb)_2_]^4+^ induces cancer cell apoptosis as well as its in vivo study as an anticancer drug.

## Conclusions

DNA polymorphism exerts a fascination on a large scientific community. It is worth mentioning that G-quadruplexes are often induced and further stabilized by the coordination of interstitial cations (e.g., Na^+^ and K^+^). This study finds that [(dmb)_2_Ru(obip)Ru(dmb)_2_]^4+^ can promote the human telomeric repeat 5′-AG3[T2AG3]3-3′ to fold into intramolecular antiparallel G-quadruplexes in the absence of Na^+^ or K^+^ cations. Clarification of the binding modes between G-quadruplex and ligand (complex) is a challenging job without crystallographic structural data. Herein, both fluorescence quenching and molecular modelling studies figure out their binding modes, through which [(dmb)_2_Ru(obip)Ru(dmb)_2_]^4+^ can strongly stabilize both the antiparallel and the mixed parallel/antiparallel G-quadruplex DNA. Moreover, [(dmb)_2_Ru(obip)Ru(dmb)_2_]^4+^ shows an obvious selectivity for G-quadruplex DNA rather than double stranded DNA and exhibits as a nanomolar potency inhibitor for telomerase. The ^tel^IC_50_ value is much lower than that of the most potent G-quadruplex interacting compounds reported in the literature. Biological function analysis demonstrated that [(dmb)_2_Ru(obip)Ru(dmb)_2_]^4+^ can significantly (P<0.05) inhibit cancer cell proliferation with minor/no side-effects to normal cells and decrease the telomerase activity of human cancer cells, implying its possibly cancer cell targeting when used as an anti-cancer drug. TUNEL assay indicated that telomerase inhibition caused genome deprotection by telomere and further DNA instability, which led to cell apoptosis. All these cellular results point to a promising candidate of [(dmb)_2_Ru(obip)Ru(dmb)_2_]^4+^ as a specific telomerase targeting anti-cancer drug even though further experiments on the underlying signaling pathways and animals models need to be performed. 

## Supporting Information

Figure S1 **CD titration of 22AG with complex in 100 mM Na^+^ or K^+^ buffer.** Titration of complex [(dmb)_2_Ru(obip)Ru(dmb)_2_]^4+^ with a 4 μM solution of G-quadruplex in 100 mM NaCl buffer (a) and in 100 mM KCl buffer (b), respectively. There are slight changes in the peaks of G-quadruplexes.
(TIF)

Figure S2 **Binding stoichiometry with G-quadruplex investigated through luminescence.** Job plot using luminescence data for [(dmb)_2_Ru(obip)Ru(dmb)_2_]^4+^ with G-quadruplex at 10 uM final using 100 mM NaCl, 10 mM NaH_2_PO_4_/Na_2_HPO_4_, 1mM Na_2_EDTA, pH=7.0, x = mole fraction of complex added to DNA.
(TIF)

Figure S3 **Molecular docking of complex and duplex DNA.** Minimized model of complex between [(dmb)_2_Ru(obip)Ru(dmb)_2_]^4+^ and duplex DNA. The G is colored in green yellow, the A is colored in red, the T is colored in cyan and [(dmb)_2_Ru(obip)Ru(dmb)_2_]^4+^ is colored in brown and blue.
(TIF)

Table S1 **MTT results analysis with GraphPad Prism5 Two-way RM ANOVA (Bonferroni posttests).**
(DOCX)
