# Supplementary figures and images for: Targeting Human Telomeric G-Quadruplex DNA and Inhibition of Telomerase Activity With [(dmb)2Ru(obip)Ru(dmb)2]4+
Source: PLoS One. 2013 Dec 27;8(12):e84419. doi: 10.1371/journal.pone.0084419 (PMC3874006; doi:10.1371/journal.pone.0084419)

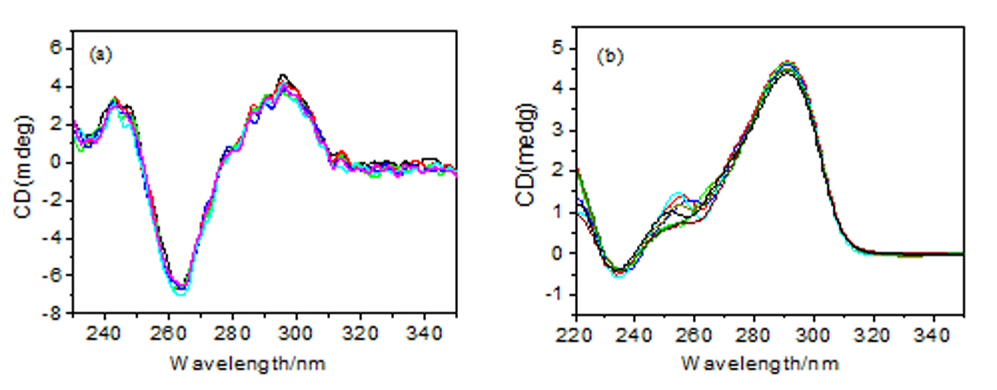

Supplement: Figure S1 — CD titration of 22AG with complex in 100 mM Na+ or K+ buffer. Titration of complex [(dmb)2Ru(obip)Ru(dmb)2]4+ with a 4 μM solution of G-quadruplex in 100 mM NaCl buffer (a) and in 100 mM KCl buffer (b), respectively. There are slight changes in the peaks of G-quadruplexes. (TIF) [file pone.0084419.s001.tif]

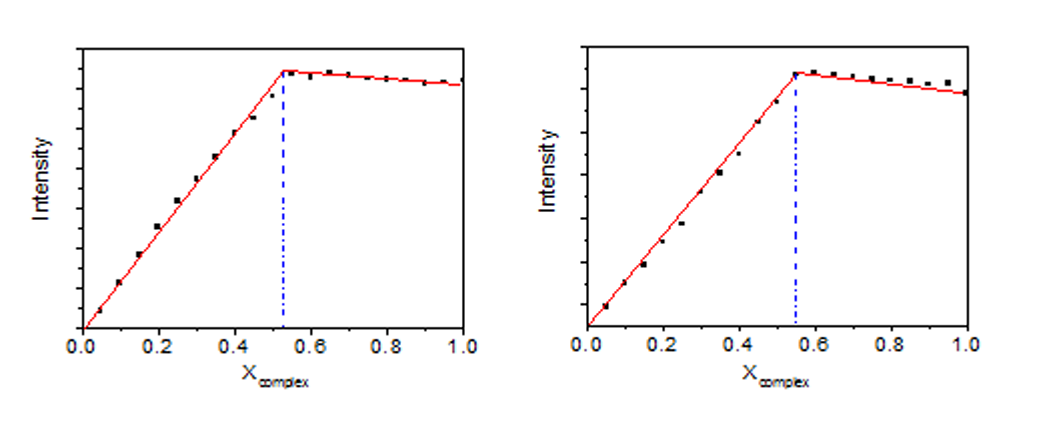

Supplement: Figure S2 — Binding stoichiometry with G-quadruplex investigated through luminescence. Job plot using luminescence data for [(dmb)2Ru(obip)Ru(dmb)2]4+ with G-quadruplex at 10 uM final using 100 mM NaCl, 10 mM NaH2PO4/Na2HPO4, 1mM Na2EDTA, pH=7.0, x = mole fraction of complex added to DNA. (TIF) [file pone.0084419.s002.tif]

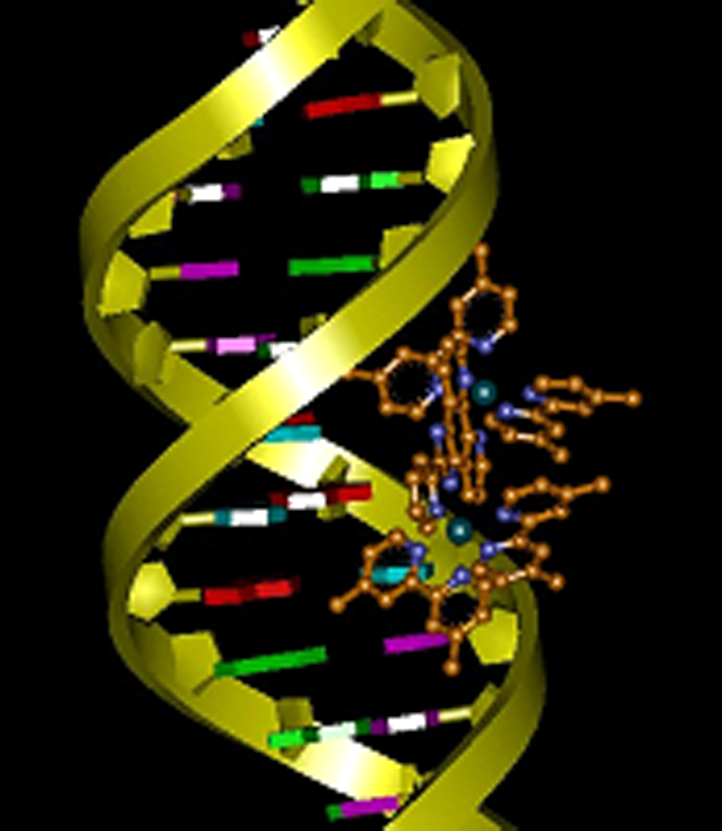

Supplement: Figure S3 — Molecular docking of complex and duplex DNA. Minimized model of complex between [(dmb)2Ru(obip)Ru(dmb)2]4+ and duplex DNA. The G is colored in green yellow, the A is colored in red, the T is colored in cyan and [(dmb)2Ru(obip)Ru(dmb)2]4+ is colored in brown and blue. (TIF) [file pone.0084419.s003.tif]
