# Supplementary material for: Targeting Human Telomeric G-Quadruplex DNA and Inhibition of Telomerase Activity With [(dmb)2Ru(obip)Ru(dmb)2]4+
Source: PLoS One. 2013 Dec 27;8(12):e84419. doi: 10.1371/journal.pone.0084419 (PMC3874006; doi:10.1371/journal.pone.0084419)
Supplement: Table S1 — MTT results analysis with GraphPad Prism5 Two-way RM ANOVA (Bonferroni posttests). (DOCX) [file pone.0084419.s004.docx]

**Table s1. MTT results analysis with GraphPad Prism5 Two-way RM ANOVA (Bonferroni posttests).**

| **Cells: Treatment Reagents** | **Time** | | **Concentration** | | **Interaction** | | **Subjects (Matching)** | |
| --- | --- | --- | --- | --- | --- | --- | --- | --- |
| P values and significant relevance | P value | sig./ns | P value | sig./ns | P value | sig./ns | P value | sig./ns |
| Fibroblast Cells: [(dmb)_2_Ru(obip)Ru(dmb)_2_]^4+^ | ˂0.0001 | *** | 0.0054 | ** | 0.7855 | ns | 0.8061 | ns |
| Hela Cells: [(dmb)_2_Ru(obip)Ru(dmb)_2_]^4+^ | ˂0.0001 | *** | 0.2972 | ns | 0.1262 | ns | 0.0804 | ns |
| Hela Cells: Cisplatin | ˂0.0001 | *** | 0.0045 | ** | 0.0154 | * | 0.5152 | ns |
| K562 Cells: [(dmb)_2_Ru(obip)Ru(dmb)_2_]^4+^ | ˂0.0001 | *** | 0.0014 | ** | 0.0084 | ** | 0.0635 | ns |
| K562 Cells: Cisplatin | ˂0.0001 | *** | 0.0012 | ** | 0.1056 | ns | 0.005 | ** |
